# Book Reviews

**Published:** 1843-03

**Authors:** 


### Bibliographical Notices.

*Treatise on the Dental Art, &c. illustrated by two hundred and forty-one figures in Lithography and fifty-four wood cuts.* By F. MAURY, dentist of the Royal Polytechnic School. Translated from the French, with notes and additions. By J. B. SAVIER, Doctor of Dental Surgery. PHILADELPHIA; LEA & BLANCHARD, pp. 293.

WE announced in the last number of the Journal that a translation of the above named work was in the course of publication, by Messrs. Lea & Blanchard, and we now have the pleasure of informing our readers that it is out, and for sale in most of the book-stores. With regard to the general merits of the work, it is unnecessary to speak, having previously expressed our opinion upon the subject. We have not yet had time to read the whole of the translation, but what we have read seems to have been faithfully and correctly executed. There is less of French about it than most English translations from that language, and the members of the profession are certainly under many obligations to Dr. Savier, for the service he has thus rendered them.

The work in its new dress looks well, as do all the works that emanate from the press of the enterprising publishers, the Messrs. Lea & Blanchard, to whom we are indebted for several valuable works on odontology, and hope to have the opportunity of acknowledging our obligation to them for many more.

